# Synthesis and characterization of crosslinked polyisothiouronium methylstyrene nanoparticles of narrow size distribution for antibacterial and antibiofilm applications

**DOI:** 10.1186/s12951-016-0208-7

**Published:** 2016-07-07

**Authors:** Sarit Cohen, Chen Gelber, Michal Natan, Ehud Banin, Enav Corem-Salkmon, Shlomo Margel

**Affiliations:** Department of Chemistry, The Institute of Nanotechnology and Advanced Materials, Bar-Ilan University, 52900 Ramat Gan, Israel; The Mina and Everard Goodman Faculty of Life Sciences, The Institute for Advanced Materials and Nanotechnology, Bar-Ilan University, 52900 Ramat Gan, Israel

**Keywords:** Isothioronium methylstyrene, Dispersion polymerization, Nanoparticles, Antibacterial, Biofilm

## Abstract

**Background:**

Isothiouronium salts are well known in their variety of antimicrobials activities. The use of polymeric biocides, polymers with antimicrobial activities, is expected to enhance the efficacy of some existing antimicrobial agents, thus minimizing the environmental problems accompanying conventional antimicrobials.

**Methods:**

The current manuscript describes the synthesis and characterization of crosslinked polyisothiouronium methylstyrene (PITMS) nanoparticles (NPs) of narrow size distribution by dispersion co-polymerization of the monomer isothiouronium methylstyrene with the crosslinking monomer ethylene glycol dimetacrylate.

**Results and discussion:**

The effect of total monomer, crosslinker and initiator concentrations on the size and size distribution of the formed NPs was also elucidated. The bactericidal activity of PITMS NPs of 67 ± 8 nm diameter was illustrated for 4 bacterial pathogens: *Listeria innocua, Escherichia coli, Pseudomonas aeruginosa* and *Staphylococcus aureus.* In order to demonstrate the potential of these PITMS NPs as inhibitor of biofilm formation, polyethylene terephthalate (PET) films were thin-coated with the PITMS NPs. The formed PET/PITMS films reduced the viability of the biofilm of *Listeria* by 2 orders of magnitude, making the coatings excellent candidates for further development of non-fouling surfaces. In addition, PITMS NP coatings were found to be non-toxic in HaCaT cells.

**Conclusions:**

The high antibacterial activity and effective inhibition of bacterial adsorption indicate the potential of these nanoparticles for development of new types of antibacterial and antibiofilm additives.

**Electronic supplementary material:**

The online version of this article (doi:10.1186/s12951-016-0208-7) contains supplementary material, which is available to authorized users.

## Background

Microbial infection remains one of the most serious complications in several areas including medical devices, healthcare products, water purification systems, hospitals, dental office equipment and food packaging [1–4]. Antimicrobial activity is related to compounds that locally kill microorganisms or inhibit their growth, without being toxic to surrounding tissue. Much attention has been directed to the development of antimicrobials from both academic research and industry [5, 6]. Polymers with antimicrobial activity, polymeric biocides, are known to have enhanced efficacy compared to existing antimicrobial molecules, minimizing the environmental problems accompanying conventional antimicrobials [7]. In general, it has been reported that polycations exhibit antibacterial properties, as they interact with and disrupt bacterial or fungi cell membranes. The adsorption of polycations, which possess very high positive charge density, onto the usually negatively charged pathogenic microorganism cell surface, is enhanced as compared to monomeric cations [8]. Compared to low molecular weight biocides, polymeric biocides are non-volatile and chemically stable, along with their reduced toxicity and increased efficiency and selectivity. Furthermore, their lifetime is significantly longer and they have low penetration through the skin [9].

Thiourea, isothiouronium compounds and their derivatives constitute an important class of compounds which exhibit wide range of antimicrobial activities and play important roles in many chemical and biological processes [10–13]. The role of the terminal amino group, belonging to the isothioronium group, seems to consist mainly of favoring the binding with peptide terminating acyl-d-alanyl-d-alanine [14]. Trani et al. [15] reported that the enhanced activity of isothiouronium compounds, compared to thiourea compounds which lack positively charged N-terminus group, may be due to the enhanced acidity of the NH moieties, thereby functioning as a better binder to Ac-d-Ala-d-Ala, a bacterial cell-wall model (tenfold lower binding constant).

Adhesion and subsequent growth of bacteria on surfaces cause the formation of biofilm. There is a growing demand for reliable antibacterial surfaces that can effectively minimize bacterial colonization [16]. Quaternary ammonium salts are widely used as ‘cationic disinfectants’ or biocidal coating to minimize the problems of biofouling [17, 18].

Listeria monocytogenes, a Gram-positive facultative anaerobe, ubiquitous in nature and common in foods of both plant and animal origin has emerged into a highly problematic and fatal foodborne pathogen throughout the world. An effective method of preserving foods from the effect of microbial growth, such as adding antimicrobial agents, would reduce the likelihood of foodborne outbreaks of listeriosis, and decrease economic losses to the food industry [5, 19, 20].

Vinylic monomers are routinely used for preparing organic polymeric NPs since they may contain many possible functional side groups [21, 22]. The present manuscript describes the synthesis and evaluation of the antimicrobial activity of novel polyisothioronium methylstyrene (PITMS) nanoparticles (NPs), by dispersion co-polymerization of the monomer isothioronium methylstyrene (ITMS) and the crosslinking monomer ethylene glycol dimethacrylate (EGDMA) in an aqueous continuous phase [18, 19]. The antimicrobial activity of the obtained PITMS NPs was evaluated using 4 bacterial pathogens: *Listeria innocua, Escherichia coli* (*E. coli*)*, Pseudomonas aeruginosa* (*P. aeruginosa*) and *Staphylococcus aureus* (*S. aureus*). The potential use of these PITMS NPs as an inhibitor of biofilm formation was demonstrated with *Listeria* as a model.

## Results and discussion

### Synthesis and characterisation of PITMS NPs

**Fig. 1:**
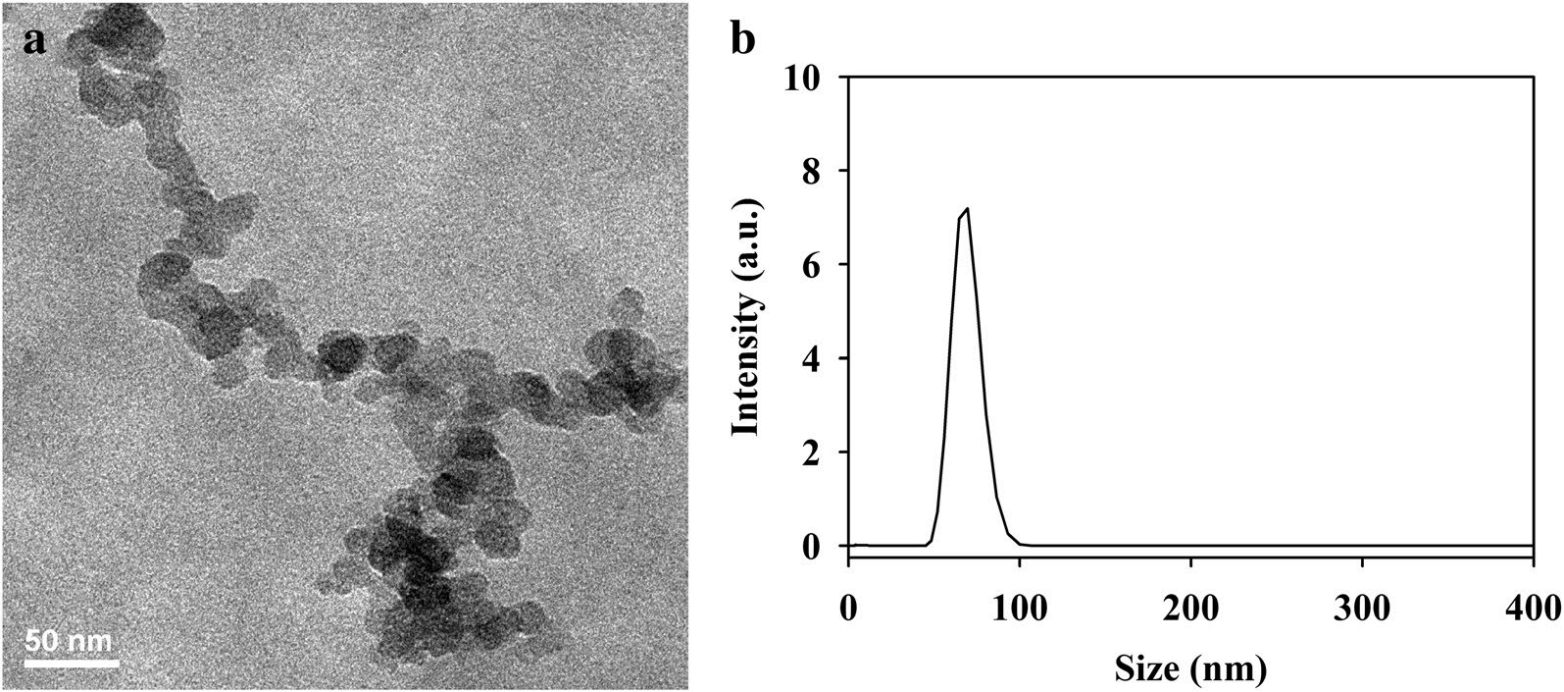
TEM image (**a**) and hydrodynamic size histogram (**b**) of the PITMS NPs

PITMS NPs of narrow size distribution were prepared by dispersion co-polymerization of ITMS and EGDMA according to the experimental part. The polymerization yield of the obtained PITMS NPs was calculated to be 75 %. Figure 1 presents a TEM image (Fig. 1a) and a typical hydrodynamic size histogram (Fig. 1b) of the obtained PITMS NPs. The dry diameter and size distribution of these PITMS particles, as shown by the TEM image, are 19 ± 2 nm, while the hydrodynamic diameter and size distribution of these particles dispersed in water, as shown by the size histogram, are 67 ± 8 nm. The hydrodynamic diameter is larger than the dry diameter probably since it also takes into account swollen and surface-adsorbed water molecules.

**Fig. 2:**
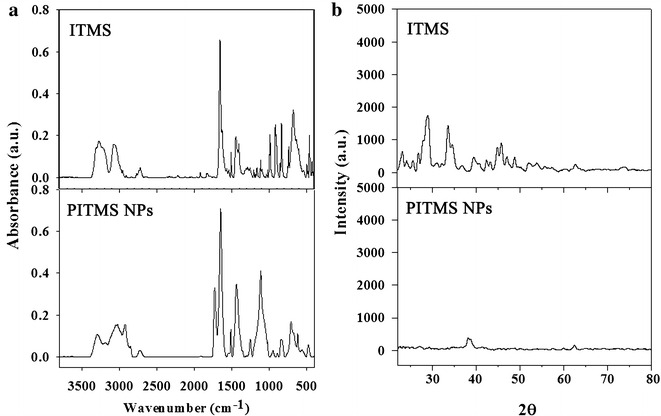
FTIR spectra (**a**) and X-ray diffraction patterns (**b**) of the ITMS monomer and the PITMS NPs

FTIR spectra of the ITMS monomer and the PITMS NPs are shown in Fig. 2a. The FTIR spectrum of the PITMS NPs is similar to that of the monomer, except for the additional absorption peak at about 916 and 987 cm^−1^ corresponding to the vinylic C–H bending band indicating the lack of residual monomer within the polymeric particles. Instead, the peaks that appears at 1110 and 1730 cm^−1^ corresponding to the C–O and C=O stretching band of EGDMA.

X-ray diffraction patterns of the ITMS monomer and the PITMS NPs are illustrated in Fig. 2b. The XRD pattern of the ITMS monomer displays clear sharp and narrow diffraction peaks typical for crystalline materials. These X-ray diffraction patterns indicate the crystalline nature of the monomer. In contrast, the X-ray diffraction pattern of the PITMS NPs, suggests the existence of a fully amorphous phase of the polymer, probably due to the loss of the crystalline structure of the monomer by the radical polymerization process.

**Fig. 3:**
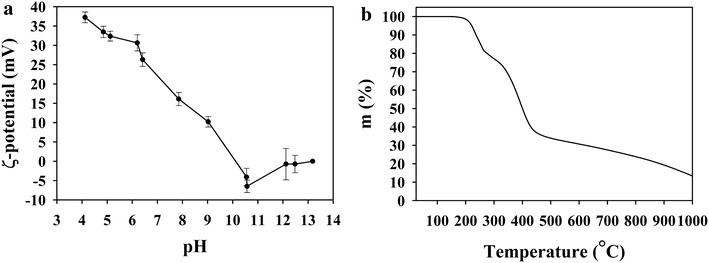
ζ-potential as a function of pH (**a**) and TGA thermogram (**b**) of the PITMS NPs

The stability of the nanoparticle dispersion was evaluated by their ζ–potential, as shown in Fig. 3a. Since a positive particle surface charge will create repulsion between the particles and may prevent aggregation, the ζ–potential of their dispersion indicates their stability. Figure 3a illustrates a consistent sharp decrease in the ζ–potential of the nanoparticles by increasing the pH of the aqueous continuous phase from 37 mV at pH 4.0 to −6.0 mV at pH 10.5. At the isoelectric point (around pH 10.2, as shown in Fig. 3a), the particles are not stable, due to possible aggregation. Increasing the pH of the continuous phase above 11.5 probably causes hydrolysis of the isothioronium groups into deprotonated thiol groups, as reported in the literature [23].

The thermal stability is an important factor when incorporating an external substance as an additive to polymer matrices. The thermal stability of the PITMS NPs aqueous dispersion, after drying, was evaluated by TGA, as illustrated in Fig. 3b. No mass loss was observes between room temperature to 160 °C indicating the thermal stability of the NPs in this temperature range. In the range of 160–270 and 270–450 °C, mass loss of 22 and 41 % was observed, attributed to the degradation of thiourea hydrochloride and the aromatic group from the PITMS NPs, respectively, as was indicated by the MS. In the range of 450–1000 °C, mass loss of 22 % was observed, probably attributed to the degradation of the polymer crosslinked carbon chain.

### Effect of polymerization parameters on the size and size distribution of the PITMS NPs

#### Effect of the EGDMA concentration

**Fig. 4:**
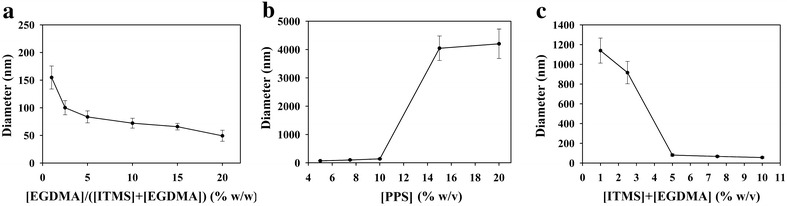
Effect of the weight ratio [EGDMA]/[ITMS + EGDMA] (**a**), initiator concentration (**b**) and total monomer concentration (**c**) on the size and size distribution of PITMS NPs

The effect of the weight ratio [EGDMA]/[ITMS + EGDMA] on the hydrodynamic diameter and size distribution of the formed PITMS NPs was studied while retaining a constant total monomer ([ITMS] + [EGDMA]) concentration (0.5 g). Figure 4a shows that as the weight ratio of [EGDMA]/[total monomers] increases the diameter and the size distribution of the formed PITMS particles decreases. For example, raising the ratio from 1 to 2.5 and 5 % leads to a decrease in the average particle size from 155 ± 21 to 100 ± 13 and 83 ± 11 nm, respectively. This behavior may be explained by the fact that increasing the crosslinking decreases the ability of the growing nuclei to swell, resulting in smaller particles [24, 25].

#### Effect of the initiator concentration

The effect of the PPS concentration on the hydrodynamic diameter and size distribution of the PITMS particles was also elucidated. Increasing the PPS concentration leads to an increase in the diameter and size distribution of the formed PITMS particles as shown in Fig. 4b. For example, raising the weight % of PPS concentration from 5 to 7.5 and 10 % leads to an increase in the average particle size and size distribution from 70 ± 8 to 100 ± 12 and 139 ± 19 nm, respectively. A similar effect of influence of the initiator concentration on the size of the particles formed by dispersion polymerization was previously reported by Tseng, El-Aasser and others [26, 27]. According to their explanation, increasing the initiator concentration causes an increase in the oligomer radical concentration, and thus, in the concentration of precipitated oligomer chain. Due to this fact, along with the slow adsorption of the stabilizer, the aggregation process is enhanced, resulting in larger particles. The increase in the size distribution as the initiator concentration increases may be explained by the increase in the number of the oligomeric chains as the initiator concentration increases, thus favoring secondary nucleation during the particle growth stage, which increases the particle size distribution.

#### Effect of the total monomer concentration

The effect of total monomers concentration ([ITMS] + [EGDMA]) on the hydrodynamic diameter and size distribution of the PITMS particles showed that increasing the total monomer concentration leads to the formation of smaller PITMS particles with narrower size distributions as shown in Fig. 4c. For example, an increase in the total monomer concentration from 2.5 to 5 and 7.5 % leads to a decrease in the diameter and size distribution of the PITMS particles from 916 ± 113 to 81 ± 12 and 67 ± 9 nm, respectively.

The decrease in the average diameter as a function of the monomer concentration was previously reported for dispersion polymerization [28, 29] although many publications have reported the opposite behavior [26, 30]. This can be explained by the effect of the monomer concentration on the dispersion polymerization mechanism. Increasing the monomer concentration may affect the initial solvency of the reaction medium by decreasing or increasing (depending on the monomer type and the continuous phase) the solubility of the forming oligomers, so they may achieve shorter or longer chain lengths before precipitating. Earlier precipitation of the shorter oligomers eventually results in a larger number of smaller particles. In addition, increasing the monomer concentration can decrease or increase the solubility of the stabilizer, thus increasing or decreasing its adsorption on the growing particle. Both effects contribute to the change in the particle size.

### Antibacterial activity of PITMS NPs

**Fig. 5:**
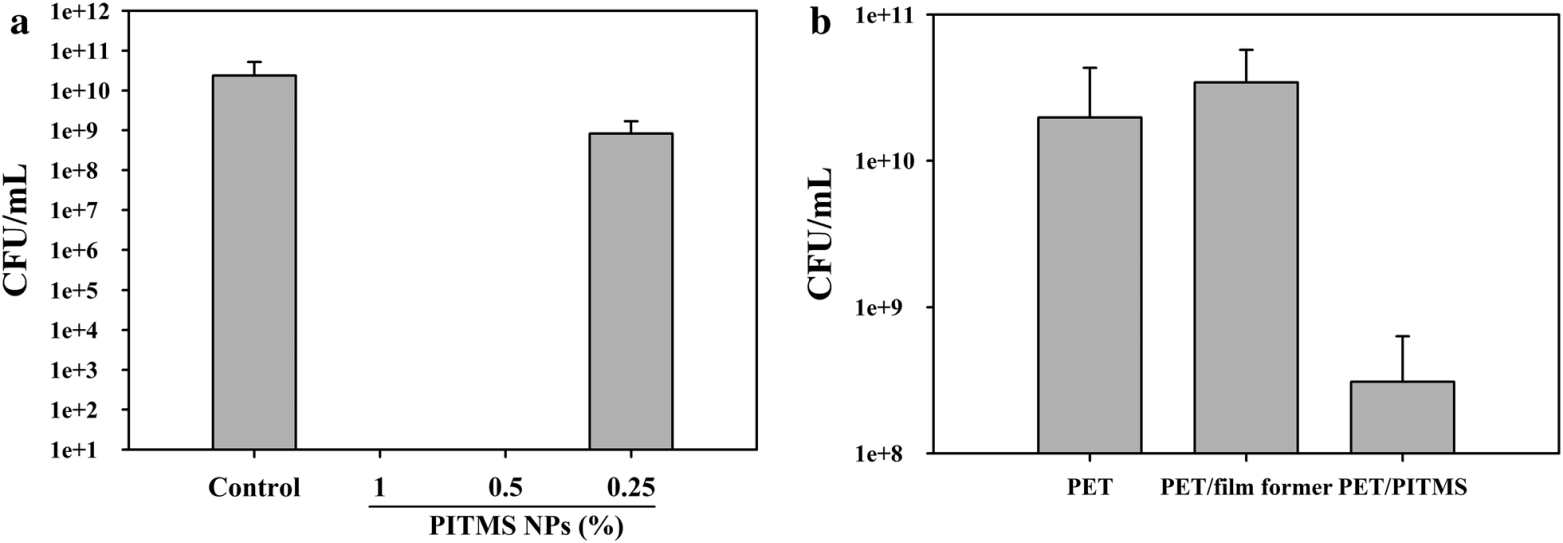
Antibacterial activity of the PITMS NPs against *Listeria* (**a**). The bacteria were grown and treated with either PITMS NPs at the indicated concentrations or water (control). The results show the pattern observed in at least three independent experiments. Biofilm formation of *Listeria* on PET films (**b**). Data is presented as the mean ± SE. The representative calculations are an average of 3 experiments, with at least tetraplicates of each experiment group in each experiment. The results of the different experiment groups in each experiment differ significantly from each other (p < 0.05), e.g. PET/PITMS NPs differ significantly from PET or PET/film former)

The antibacterial properties of PITMS NPs of 67 ± 8 nm were tested against *Listeria* bacteria, a common foodborne pathogen. As shown in Fig. 5, both 1 and 0.5 % PITMS NPs were able to kill all the tested bacteria following 24 h exposure, as opposed to the water-treated control, suggesting a potent antimicrobial activity of the PITMS particles. A 0.25 % particle concentration had only a partial bactericidal effect, implying that 0.5 % is the minimum inhibitory concentration (MIC) for PITMS needed to inhibit growth under these experimental conditions. Similar results were obtained for three additional pathogenic bacteria: *E. coli, P. aeruginosa* and *S. aureus* as shown in the Additional file 1: Figure S1.

### Antibiofilm activity of the PITMS NPs

In light of the antibacterial activity exerted by the PITMS, the inhibition of *Listeria* biofilm formation by the NPs was determined. As shown in Fig. 5b, a significant reduction in the biofilm formation of *Listeria* was detected; 2 logs for the PET/PITMS films, in comparison to a film containing the film former only or to a non-coated PET film.

### Migration tests

Migration is the term used for the escape of additives from a polymeric host. Migration may limit the use of additives in plastic especially for food packing applications, pharmaceutical and other hygienic products. Crosslinked NPs that are compatible with the PET film and the film former may overcome this disadvantage, due to their large spatial structure, which reduces their migration while maintaining the activity. Hence, using appropriate NPs as an additive to plastic films will result in antibacterial properties and with decreased extractability and volatility. Indeed, no migration of the PITMS NPs to the continuous phase composed of 3 % aqueous acetic acid or 95 % ethanol were detected.

### Optical properties of the PET/PITMS films

The optical properties of polymeric films are important for many applications, such as transparent food packaging. The haze, clarity and transmittance of the PET/PITMS in comparison to a film containing the film former only or to a non-coated PET film are shown in Table 1. The results indicate the potential use of the PET/PITMS films as transparent films. There was no change in the optical properties of the various films after a year indicating that there is no migration during this time period.

**Table 1:** Haze, clarity and transmission of the PET, PET/film former and PET/PITMS films

	Haze (%)	Clarity (%)	Transmission (%)
PET	1.3 ± 0.1	99.2 ± 0.0	89.5 ± 0.1
PET/film former	2.5 ± 0.2	98.9 ± 0.04	90.6 ± 0.5
PET/PITMS	6.5 ± 0.4	94.5 ± 0.7	91.0 ± 0.1

### Cellular cytotoxicity of PITMS NP coatings by LDH assay

**Fig. 6:**
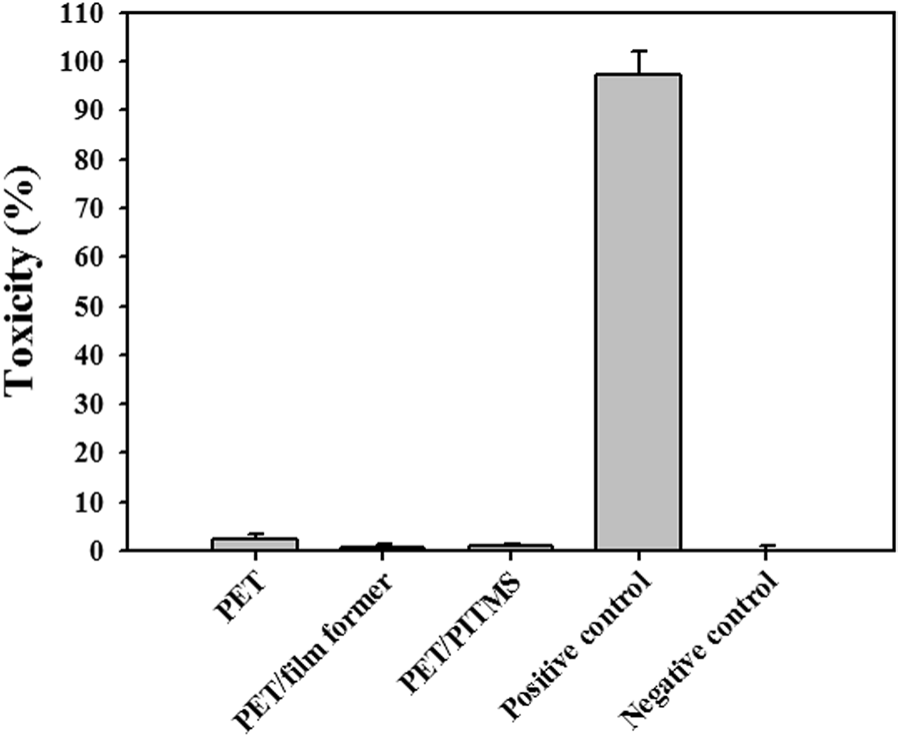
Cytotoxic effect of the PITMS NP coatings on HaCaT cells measured by the LDH assay. Cells (3 × 10^5^) were incubated with the supernatant of the various films that were pre-incubated in the medium according to the experimental section. Cells were incubated with Triton-x-100 1 % as positive control (100 % toxicity). Untreated cells (negative control) were similarly incubated. Each bar represents mean ± SE of 4 separate samples

In vitro cytotoxicity of the PITMS NP coatings was tested by LDH assay using human keratinocyte HaCaT cells [31]. HaCaT cell line is a spontaneously transformed human epithelial cell line from adult skin and the first permanent epithelial cell line that exhibits normal differentiation [32]. Lactate dehydrogenase, LDH, is an intracellular enzyme that catalyzes the reversible oxidation of lactate to pyruvate. Since LDH is mainly present in the cytosol, it is released into the supernatant only upon cell damage or lysis [33, 34]. When tested by the LDH quantitative assay, all the pre-incubated PET films had no cytotoxic effect on the HaCaT cell line (Fig. 6). PET/PITMS films are therefore suitable for food application, considering their non-toxicity.

## Conclusions

The present study describes the synthesis and characterization of PITMS NPs of narrow size distribution. The effect of various polymerization parameters on the size and size distribution of the produced PITMS particles have been elucidated. This study also demonstrates that these NPs have excellent antibacterial activity against *Listeria*. This work integrates the advantages of polymer chemistry and technology with bacteriology, leading to possible developments in the formulation of new types of bactericides. The PET/PITMS films under the experimental conditions inhibited biofilm formation of *Listeria* by 2 orders of magnitude, making the coatings excellent candidates for further development of non-fouling surfaces. In future work we plan to check the antibiofilm activity of the NPs against additional types of bacteria and additional types of films in various coating thicknesses.

## Methods

### Materials

The following analytical-grade chemicals were purchased from Sigma-Aldrich (Israel) and used without further purification: thiourea, chloromethylstyrene (CMS; 97 %), potassium persulfate (PPS), ethylene glycol dimetacrylate (EGDMA), methanol, diethyl ether and tween 20. PET films of A4 size and 23 µm thick were obtained from Hanita Coatings RCA Ltd, Israel. Film former G-9/230 from ACTEGA Coating & Sealants, Wesel, Germany. Water was purified by passing deionized water through an Elgastat Spectrum reverse osmosis system (Elga Ltd, High Wycombe, UK). Dulbecco’s minimum essential medium (DMEM) eagle, fetal bovine serum (FBS), 1 % glutamine, 1 % penicillin/streptomycin and mycoplasma detection kit from Biological Industries (Bet Haemek, Israel); cytotoxicity detection kit from BioVision, USA; the HaCaT cell line was a kind gift from Prof. Eli Shprecher, Molecular dermatology research laboratory, Tel-Aviv Sourasky medical center, Israel, and was cultured as previously described.

### Synthesis of the isothioronium methylstyrene monomer

**Fig. 7:**
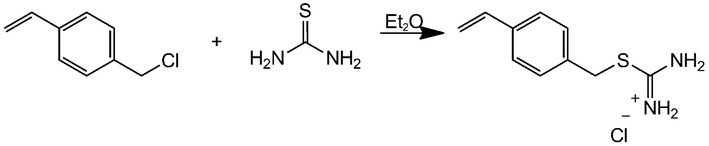
Synthetic scheme for the ITMS monomer

The monomer ITMS was synthesized according to the literature, as shown in Fig. 7. Briefly, thiourea (0.21 mol) was dissolved in methanol (60 mL), followed by the addition of *p*-chloromethylstyrene (CMS, 0.2 mol) to the solution. The reaction mixture was then stirred at room temperature for 24 h. Diethyl ether was then added to precipitate the desired ITMS monomer. The filtered product was purified by dissolving it in ethanol and re-precipitation with ether (41.13 g, 0.18 mol, 90 %). The solid residue was analyzed by ^1^H and ^13^C NMR which showed the pure desired product.

Nuclear magnetic resonance (NMR) spectroscopy was performed on Bruker AC 400 MHz spectrometer. ^1^H and ^13^C NMR spectra were recorded in deuterated dimethyl sulfoxide.

^1^H NMR (400 MHz, DMSO-*d*_*6*_) δ_H_ in ppm: 4.53 (s, 2H, CH_2_–S), 5.28 (d, 10.8 Hz, 1H, CH_2_=CH [trans]), 5.85 (d, 17.6 Hz, 1H, CH_2_=CH [cis]), 6.73 (dd, 10.8 Hz and 17.6 Hz, 1H, CH_2_=CH [gem]), 7.41 (d, 8.2 Hz, Arom-CH), 7.48 (d, 8.2 Hz. Arom-CH), 9.32 (s, 4H, Isothiouronium).

^13^C NMR (400 MHz, DMSO-*d*_*6*_) δ_C_ in ppm: 34.8 (CH_2_–S), 114.9 (**C**H_2_=CH), 126.6 and 129.1 (Arom-CH), 133.6 and 135.8 (Arom C), 137.4 (CH_2_=**C**H), 170.3 (C-Isothiouronium).

MS (CI+): 117 (CH_2_CHArCH_2_^+^, 100 %), 193 (M+, 10.5 %).

### Synthesis of the polyisothioronium methylstyrene NPs

In a typical experiment, PITMS NPs with dry diameter of 67 ± 8 nm were prepared by adding ITMS (425 mg), EGDMA (75 mg), PPS (25 mg) and Tween 20 (100 mg) to water (10 mL). The mixture was shaken at 73 °C for 15 h. The obtained NPs dispersed in water were then isolated from impurities by dialysis. The formed PITMS particles were washed with water at 60 °C in order to remove traces of the monomers and excess reagents. The effect of various polymerization parameters, e.g., total monomers, initiator and crosslinker concentrations, on the size and size distribution, and the polymerization yield of the ITMS to produce the particles was also elucidated.

### Coating of the PET films with the PITMS NPs

PET films were coated with the NPs aqueous dispersion using a formulation with a ratio of 1:1 PITMS polymer to the film former (G-9/230). PITMS NPs of 67 ± 8 nm diameter dispersed in water (4 %) were first dispersed in the G-9/230 film former 4 % aqueous solution (1:1 v/v). The obtained aqueous dispersion was then spread on the 23 μm thick PET films with a 6 μm (wet thickness) Mayer rod, followed by drying the PITMS coating on the PET films over night at room temperature.

### Antibacterial assay

The antibacterial activity of the PITMS NPs of 67 ± 8 nm diameter was evaluated using the Gram-negative bacteria *E. coli* C600 and *P. aeruginosa* PAO1, and the Gram-positive bacteria *S. aureus* FRF1169 and *Listeria* ATCC 33,090, as the experimental models. All the bacterial strains used in this study were grown overnight in Luria Bertani (LB, Difco) media under shaking (250 rpm) at 37 °C. On the following day, the overnight cultures were each diluted into twofold concentrated LB medium to obtain a concentration of 2 × 10^5^ colony-forming units (CFU/mL). The bacterial suspensions were incubated overnight with equivalent volumes of either PITMS (1, 0.5 and 0.25 %) or sterilized water (control). In the following day, tenfold serial dilutions were carried out and the bacterial cells were plated on LB agar plates, followed by their incubation at 37 °C for 20 h. Cell growth was monitored and determined by viable cell count and expressed as colony forming units (CFU/mL).

### Static biofilm formation assay

The antibiofilm activity of the PET/PITMS films against *Listeria* bacteria was evaluated according to a protocol that was previously published [35] and compared to a control PET film and a PET film coated with the film former only using the Gram-positive bacteria *Listeria* ATCC 33,090 as the experimental model. Briefly, the bacteria were grown overnight in tryptic soy broth (TSB, DIFCO) growth medium. In the following day, bacterial cells were diluted in TSB to obtain a working solution with an OD_595_ of 0.3 (approximately corresponds to 3 × 10^8^ CFU/mL). 1 mL from the stock solution was taken into each well in a 24-well plate (DE-GROOT). Each of the different films was added to the well (1 cm diameter). The plates were then incubated at 25 °C under gentle agitation (100 rpm) for 20 h. In the day after, the films were rinsed 3 times with distilled water to remove the unattached bacteria (i.e. planktonic cells) and subsequently the attached cells were scraped from the films using 250 µl of Tris–HCl (0.1 M, pH 7.2) and cell scrapers (Greiner Bio-one). 200 µl out of the 250 µl, used for scrapping the cells, were transferred into the first line of a 96-well plate (Greiner Bio-One), while the rest of the lines were filled with 180 µl of Tris–HCl (0.1 M, pH 7.2). Serial dilutions were carried out and the cells spotted onto NB agar plates, which were then incubated at 37 °C for 20 h. Cell growth was monitored and determined by a viable cell count. The experiments were conducted at least three independent times, with internal duplicates.

### Characterization of the PITMS NPs and the PET/PITMS films

Fourier transform infrared (FTIR) analysis was performed with a Bruker Platinum-FTIR QuickSnap TM sampling modules A220/D-01. The analysis was performed with 13 mm KBr pellets that contained 2 mg of the detected material (ITMS or PITMS) and 198 mg KBr. The pellets were scanned over 50 scans at a 4 cm^−1^ resolution.

Electrokinetic properties (ζ-potential) as a function of pH were determined with Zetasizer (Zetasizer 3000 HSa, Malvern Instruments, UK). ζ-potential measurements were performed at a constant ionic strength of 0.1 M.

Dried particle size and size distribution were measured with a transmission electron microscope (TEM). SEM pictures were obtained with a JEOL, JSM-840 Model, Japan. For this purpose, a drop of dilute particles dispersion in distilled water was spread on a glass surface, and then dried at room temperature. The dried sample was coated with carbon in vacuum before viewing under SEM. The average particle size and distribution were determined by the measurement of the diameter of more than 100 particles with image analysis software (Analysis Auto, Soft Imaging System GmbH, Germany).

Hydrodynamic diameter and size distribution of the particles dispersed in double distilled (DD) water were measured at room temperature with a particle analyzer; model NANOPHOX (SympatecGmbH, Germany).

The thermal behavior of the PITMS NPs was determined by thermo gravimetric analysis (TGA) with a TA TGA Q500 instrument combined with mass spectrometer (MS) from Thermo-star Pfeiffer Inc.

The weight % polymerization yield of the ITMS to form PITMS NPs was calculated by the following expression:

$${\text{Polymerization}}\;{\text{yield }}\left( {{\text{weight}}\,\% } \right) = \left[ {{\text{W}}\left( {\text{PITMS}} \right)/{\text{W}}\left( {{\text{ITMS}} + {\text{EGDMA}}} \right)} \right]\, \times \,100$$

where W(PITMS) is the weight of the dried PITMS NPs and W(ITMS + EGDMA) is the initial weight of the ITMS and EGDMA monomers.

Film thicknesses were measured on a Millitron 1204 IC (Mahr Feinmesstechnik GmbH). The optical parameters transmittance, haze, and clarity of the films were measured on a BYK Gardner haze-gard plus in accordance with ASTM D1003 “Standard Test Method for Haze and Luminous Transmittance of Transparent Plastics”. The PET, PET/film former and PET/PITMS films are irradiated with visible light; the transmitted intensity is then integrated by the instrument. Haze and clarity are per definition components of scattered light under wide angle (>2.5°) and narrow angle (<2.5°), respectively. Mean values and standard deviations of transmittance, haze, and clarity were obtained by taking the average over several measurements (at least 4 measurements each).

### Migration test

A specimen of 0.5 dm^2^ of each film (PET, PET/film former and PET/PITMS films) was incubated with 50 mL of 3 % acetic acid in distilled water or 95 % ethanol for 2 h at 70 °C. The migration of the NPs from the PET/PITMS films into the continuous phase was accomplished by weighing the PET/PITMS films before and after the incubation and measuring the absorbance spectrum of the filtrate using Carry 100 UV–VIS spectrophotometer (Agilent Technologies Inc.) at a range of 200–600 nm. In addition, the haze of the PET/PITMS films during time was also measured.

### Cytotoxicity of the PITMS NP coating

In vitro cytotoxicity of the PITMS NP coatings was tested by using HaCaT cell line. The cell line is adherent to the used culture dishes. HaCaT cells were grown in DMEM-eagle that was supplemented with 10 % heat-inactivated fetal bovine serum (FBS), 1 % glutamine and 1 % penicillin/streptomycin. Cytotoxicity was performed in two steps. First, the PET, PET/film former and PET/PITMS films were incubated within the medium at 37 °C for 24 h in a humidified 5 % CO_2_ incubator. The next step is incubation of the supernatant with HaCaT cell line at 37 °C for 48 h and then measuring the release of cytoplasmic lactate dehydrogenase (LDH) into the cell culture supernatants.

Cell cytotoxicity was assessed by measuring the release of LDH into cell culture supernatants. LDH activity was assayed using the Cytotoxicity Detection Kit according to the manufacturer’s instructions. Cells (3 × 10^5^ cells per well) were seeded and grown to 75–80 % confluency in 96 well plates before treatment with the films supernatants. Cell cultures that were not exposed to the films supernatants were included in all assays as negative controls. Cell cultures that were treated with 1 % Triton-x-100 were used as positive controls. The cell cultures were further incubated at 37 °C in a humidified 5 % CO_2_ incubator and then checked for cellular cytotoxicity after of 48 h. The percentage of cell cytotoxicity was calculated using the formula shown in the manufacturer’s protocol [33]. All samples were tested in tetraplicates.

## Additional file

10.1186/s12951-016-0208-7 Antibacterial activity of the PITMS NPs against *E. coli*, *P. auruginosa* and *S. aureus*. The three bacterial strains were grown and treated with either PITMS NPs at the indicated concentrations or water (control).

## Abbreviations

CFU: colony-forming units
CMS: *p*-chloromethylstyrene
DD: double distilled
DMEM: Dulbecco’s minimum essential medium
*E. coli*: *Escherichia coli*
EGDMA: ethylene glycol dimethacrylate
FBS: fetal bovine serum
FTIR: Fourier transform infrared
ITMS: isothiouronium methylstyrene
LB: Luria–Bertani
LDH: lactate dehydrogenase
MIC: minimum inhibitory concentration
MS: mass spectrometer
NMR: nuclear magnetic resonance
NPs: nanoparticles
*P. aeruginosa*: *Pseudomonas aeruginosa*
PET: polyethylene terephthalate
PITMS: polyisothiouronium methylstyrene
PPS: potassium persulfate
*S. aureus*: *Staphylococcus aureus*
TEM: transmission electron microscope
TGA: thermo gravimetric analysis
TSB: tryptic soy broth
