# Supplementary material for: Synthesis and characterization of crosslinked polyisothiouronium methylstyrene nanoparticles of narrow size distribution for antibacterial and antibiofilm applications
Source: J Nanobiotechnology. 2016 Jul 7;14:56. doi: 10.1186/s12951-016-0208-7 (PMC4936196; doi:10.1186/s12951-016-0208-7)
Supplement: Supplementary file 1 — 10.1186/s12951-016-0208-7 Antibacterial activity of the PITMS NPs against E. coli, P. auruginosa and S. aureus. The three bacterial strains were grown and treated with either PITMS NPs at the indicated concentrations or water (control). [file 12951_2016_208_MOESM1_ESM.docx]

# Supporting information

The antibacterial properties of the PITMS NPs of 67 ± 8 nm were also tested against *E. coli, P. auruginosa* and *S. aureus*, three common bacterial pathogens.

**Figure S1 Antibacterial activity of the PITMS NPs.** The three bacterial strains were grown and treated with either PITMS NPs at the indicated concentrations or water (control).
